# Development under predation risk increases serotonin-signaling, variability of turning behavior and survival in adult fruit flies *Drosophila melanogaster*

**DOI:** 10.3389/fnbeh.2023.1189301

**Published:** 2023-05-25

**Authors:** Tatjana Krama, Māris Munkevics, Ronalds Krams, Tatjana Grigorjeva, Giedrius Trakimas, Priit Jõers, Sergejs Popovs, Krists Zants, Didzis Elferts, Markus J. Rantala, Eriks Sledevskis, Jorge Contreras-Garduño, Benjamin L. de Bivort, Indrikis A. Krams

**Affiliations:** ^1^Department of Biotechnology, Institute of Life Sciences and Technologies, Daugavpils University, Daugavpils, Latvia; ^2^Chair of Plant Health, Estonian University of Life Sciences, Tartu, Estonia; ^3^Department of Zoology and Animal Ecology, Faculty of Biology, University of Latvia, Riga, Latvia; ^4^Institute of Biosciences, Vilnius University, Vilnius, Lithuania; ^5^Institute of Molecular and Cell Biology, University of Tartu, Tartu, Estonia; ^6^Department of Botany and Ecology, Faculty of Biology, University of Latvia, Riga, Latvia; ^7^Department of Biology, Turku Brain and Mind Center, University of Turku, Turku, Finland; ^8^Department of Technology, Institute of Life Sciences and Technologies, Daugavpils University, Daugavpils, Latvia; ^9^Escuela Nacional de Estudios Superiores, Universidad Nacional Autónoma de México, Morelia, Mexico; ^10^Institute for Evolution and Biodiversity, University of Münster, Münster, Germany; ^11^Department of Organismic and Evolutionary Biology, Harvard University, Cambridge, MA, United States; ^12^Latvian Biomedical Research and Study Centre, Riga, Latvia; ^13^Institute of Ecology and Earth Sciences, University of Tartu, Tartu, Estonia; ^14^Department of Psychology, University of Tennessee, Knoxville, Knoxville, TN, United States

**Keywords:** *Drosophila melanogaster*, behavioral predictability, serotonin, survival under predation, turning behavior

## Abstract

The development of high-throughput behavioral assays, where numerous individual animals can be analyzed in various experimental conditions, has facilitated the study of animal personality. Previous research showed that isogenic *Drosophila melanogaster* flies exhibit striking individual non-heritable locomotor handedness. The variability of this trait, i.e., the predictability of left-right turn biases, varies across genotypes and under the influence of neural activity in specific circuits. This suggests that the brain can dynamically regulate the extent of animal personality. It has been recently shown that predators can induce changes in prey phenotypes via lethal or non-lethal effects affecting the serotonergic signaling system. In this study, we tested whether fruit flies grown with predators exhibit higher variability/lower predictability in their turning behavior and higher survival than those grown with no predators in their environment. We confirmed these predictions and found that both effects were blocked when flies were fed an inhibitor (αMW) of serotonin synthesis. The results of this study demonstrate a negative association between the unpredictability of turning behavior of fruit flies and the hunting success of their predators. We also show that the neurotransmitter serotonin controls predator-induced changes in the turning variability of fruit flies, regulating the dynamic control of behavioral predictability.

## Introduction

Living organisms adapt to varying environmental conditions by attempting to modify their morphological, biochemical, and behavioral phenotypes (Xue et al., 2019). Predation has been shown to have profound lethal and non-lethal (Lima, 1998) impacts on prey individuals, affecting their behavior (Hulthén et al., 2021), phenotype development (Krams et al., 2020), fitness (Zanette and Clinchy, 2020; Allen et al., 2022), population structure and evolution (Yartsev, 2017; Dudeck et al., 2018). Prey individuals respond to predator acoustic, visual, chemical, and other cues, which improve the chances of prey to escape predator attacks (Lima, 1998; Preisser et al., 2005; Peckarsky et al., 2008; Voelkl et al., 2016; Zanette et al., 2019; Krams R. et al., 2021). When developing under predation risk, prey individuals often grow smaller, more agile, less palatable, or more cryptic, conferring fitness benefits associated with a modified phenotype (Krams et al., 2016). Fruit flies (*Drosophila melanogaster*) raised during the larval stage together with jumping spiders had more nitrogen in their bodies and lower body lipid reserves, while they had a higher climbing speed in the negative geotaxis test than flies grown without spiders (Krams et al., 2016). Moreover, fruit flies grown together with predators had significantly higher adult survival ability when exposed to predation than flies grown in a predator-free environment (Krams et al., 2016). This shows that predator exposure in ontogeny may directly affect survival in adulthood. However, it is not always clear what changes in the neural and behavioral phenotypes facilitate the escape performance of fruit flies at risk of predation.

Locomotor activity has been shown to change adaptively during the evolutionary “arms race” between prey and predator by enhancing the predator escape ability of prey individuals (Moore and Biewener, 2015; Moore et al., 2017). Jumping spiders are ambush predators whose attacking repertoire involves direct attacks triggered by the approaching prey (Rößler et al., 2022). Ambush predators remain concealed and motionless until the prey comes within ambush distance before pouncing. If the prey survives in the initial attack, the predator often does not pursue it (Scharf et al., 2006). Although ambush predators are not supposed to actively rely on predictions of the prey’s behavior (Caraco and Gillespie, 1986; Mischiati et al., 2015; Moore and Biewener, 2015), the lower predictability of approach trajectories of prey may affect the chances of the prey to approach the predator’s ambush distance. Moreover, so-called “protean” behavior is defined as a sufficiently unpredictable response to prevent a predator from anticipating its prey’s future position or actions (Humphries and Driver, 1970; Richardson et al., 2018). However, the exact predictability of potentially “protean” prey behaviors has received limited observational and experimental attention.

Fruit flies exhibit striking locomotor handedness during their exploratory behavior (Buchanan et al., 2015; de Bivort et al., 2022), one example of the preferential performance of a behavior on one side of the body. During exploratory walking in symmetrical environments, individual fruit flies exhibit significant bias in their left vs. right/counter-clockwise vs. clockwise locomotor choices, with some flies being strongly left-biased or right-biased. This behavioral idiosyncrasy is present across different fly lines and genotypes. Moreover, the flies differing in neural state (Buchanan et al., 2015) or genotype (Ayroles et al., 2015) differed in the extent of left vs. right turning bias. Specifically, the magnitude of turning bias variation is under the control of columnar neurons within the central complex, a brain region implicated in motor planning and execution of fruit fly behavior (Buchanan et al., 2015). Turn bias variability has a complex genetic architecture involving many genes, particularly those involved in circuit development during pupation, including specifically *teneurin-A* (Ayroles et al., 2015) that encodes a protein involved in synaptic partner matching (Mosca, 2015). Silencing the central complex columnar neurons or knocking down *teneurin-A* expression increased exploratory laterality in fruit fly turning behavior, with more extreme leftiness and rightiness, decreasing the predictability of turning choices across individuals. In the mathematical limit, a population with maximal turn bias unpredictability across individuals (composed of equal parts extreme righties and extreme lefties) would consist of animals with high within-individual predictability (making exclusively right or left turns, respectively). But in experiments, examining microhabitat occupancy, a positive correlation was observed between population-level behavioral predictability and individual predictability (Stamps et al., 2013). In this study, we use “predictability” to refer to variability in behavior at the population level, across individuals.

Neurotransmitters are known to control the predictability of behavior (Maloney, 2021). The predictability of phototaxis in flies is under the control of the neurotransmitter serotonin (5-HT), and the lowest predictability of turning choices were found in white-eyed *w*^1118^ mutants (Kain et al., 2012; Krams I. A. et al., 2021). White-eyed flies have 32% less 5-HT in their heads than the brains of red-eyed fruit flies (Borycz et al., 2008; Krstic et al., 2013). 5-HT also regulates the predictability of odor preferences in flies (Honegger et al., 2020) and locomotor activity in the roundworm *C. elegans* (Stern et al., 2017; Nasser et al., 2022). With respect to turn bias variability, both increasing and decreasing 5-HT with metabolic drugs had small effects of reducing turn bias variability, averaged across many genotypes (de Bivort et al., 2022). Applying serotonin precursor increases variability in locomotor speed, and there is a bidirectional effect of altering serotonin levels on variability in higher-order left-right turn sequences (de Bivort et al., 2022). All these effects are small, but they generally suggest a role for serotonin in decreasing locomotor predictability. It has been recently shown that predator-induced stress influences a number of 5-HT-associated behavioral and physiological effects in fruit flies grown together with spiders during larval development (Krama et al., revision 2, personal observation). This implies that predators may influence the brain to dynamically regulate the predictability of the turning behavior of fruit flies to improve their survival under predation risk.

In this study, we tested whether fruit flies reared with spiders exhibit lower predictability in their turning behavior in Y-mazes (Buchanan et al., 2015), compared to flies reared in predator-free environments. We also studied the survival of fruit flies grown with and without spiders. To investigate the role of 5-HT in regulating antipredator behavior, we fed fruit flies raised with spiders and flies raised without spiders 5-HTP (a precursor of 5-HT) and αMW (a serotonin-synthesis inhibitor). We hypothesized that predator presence during larval development might make the turning behavior of adult fruit flies less predictable and improve their survival. We predicted that feeding αMW might make turning choices of flies reared together with predators more predictable (de Bivort et al., 2022) and decrease their survival. We studied male fruit flies only because a large portion of the body of a mated/unmated female is composed of developing eggs and reproduction-related tissue, which may influence body mass, body size, metabolism, and antipredator behavior, potentially affecting predator preferences (Burggren, 2017). Individual-to-individual differences in experimental behavioral observations reflect persistent idiosyncrasies requiring large samples (Sih et al., 2004; Mollá-Albaladejo and Sánchez-Alcañiz, 2021). Small mazes arrayed in parallel allow the measurement of behavior of hundreds of individual flies simultaneously (de Bivort et al., 2022) and high-powered inference of the effects of experimental manipulations (Brown and de Bivort, 2018).

## Materials and methods

### Prey, predators, developmental speed, and the main treatment groups

The wild type strain of *D. melanogaster* [Oregon-R-modENCODE (#25211)] was obtained from Bloomington Drosophila Stock Center (IN, US). This line of OR was inbred for 10 generations before behavioral experiments were collected. We reared our stock flies at the University of Tennessee-Knoxville at 24 ± 1°C, at 40% relative humidity with a 12/12 h light/dark cycle.

We used adult jumping spiders (*Phidippus apacheanus*) as predators to affect the development and behavior of fruit flies. *P. apacheanus* is widely distributed across the US, and the spiders are easy to maintain in the lab because they readily consume both larvae and adults of *D. melanogaster* (Krams et al., 2016). The adult spiders were caught in Florida and delivered by the supplier phids.net.

Developmental speed significantly affects body mass, elemental body composition, food uptake, and fat metabolism of *D. melanogaster* (Krams et al., 2020). This makes the flies with rapid, intermediate, and slow development different in their biochemical and morphological phenotype, which needs to be considered when planning research. To avoid the developmental speed-related confounding effects, we used only rapidly developing fruit flies in this study. We defined rapidly developing flies as individuals that eclose between 9.5 and 10.0 days after egg-laying (Krams et al., 2020). Rapidly growing fruit flies experience relatively low stress levels during ontogeny (Krams et al., 2020).

We isolated fruit flies using carbon dioxide anesthesia within 6–7 h after eclosion. Ten F0 females and ten males were placed for 24 h in one vial (Flystuff polystyrene vials; Genesee Scientific, El Cajon, CA, USA, 24 mm inner diameter × 95 mm height) containing 6 ml of Cal Tech medium. After 24 h, the adults were removed, and the vials were placed horizontally on the floor of Plexiglas jars (10 cm height × 12 cm diameter). The density of F1 first-instar larvae across the vials was similar, and we averaged the density to 120 larvae/vial by removing extra larvae with a squirrel brush (Krams et al., 2016). Vials with *Drosophila* larvae were randomly divided into two groups: one that was exposed to spiders and one that was not. In the spider-treated group, a single *P. apacheanus* individual was also included in each Plexiglas jar. The vials did not have stoppers, giving the spider free access to the developing flies (as well as the fly media). Developing flies were also exposed to the odor of the spider throughout the container. Flies for behavioral and survival assays were removed the day after they eclosed, without anesthesia, and transferred to drug-treated vials as described below.

### Neurotransmitter treatments

**FIGURE 1:**
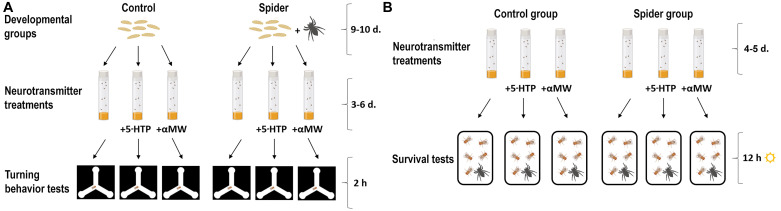
Schematic of the turning assay **(A)** and the survival experiment **(B)** (this figure was made using some sample images from BioRender.com).

We had two main experimental groups of *D. melanogaster*: flies grown together with predators and flies grown with no predators (Figure 1A); each of these two groups was further divided into three subgroups: flies raised on food supplemented with 5-HTP, flies grown on food supplemented with αMW, and flies grown without any drugs (Neckameyer, 1996; Dasari et al., 2007; Dierick and Greenspan, 2007; Majeed et al., 2016; Ries et al., 2017; Hu et al., 2020; Krams I. A. et al., 2021; Figure 1A). The drug stock solutions were vortex-mixed and added to food powder. 5-HTP and αMW were dissolved in Cal Tech instant media (United States Biological, Salem, MA, USA). The final concentration of 5-HTP was 50 mM, and the final concentration of αMW was 20 mM (Kain et al., 2012; Krams I. A. et al., 2021). The flies were 5–7 days old at the moment of behavioral experiments. Dierick and Greenspan (2007), by using HPLC, showed that 5-HTP feeding significantly increases the brain 5-HT within 3 days of treatment, while αMW significantly decreases the amount of brain 5-HT during 4 days of treatment. Honegger et al. (2020) confirmed similar effects (∼8× reduction of 5-HT with αMW treatment; ∼20× increase with 5-HTP) using ELISA assays.

### Turning behavior

Since using variance as a phenotypic trait requires large sample sizes (Caballero et al., 2021), we used a high-throughput assay to monitor the behavior of individual flies placed into individual Y-mazes (Ayroles et al., 2015; Buchanan et al., 2015). We put flies into an array containing 95 individual Y-mazes consisting of three symmetrical arms (each 12 mm long) fabricated from laser-cut acrylic (Figure 1A). Maze arrays were illuminated from below with a grid of 100 white LEDs (5500K, Knema) below acrylic diffusers. Maze arrays were imaged with 2MP digital cameras (Point Gray), and the X-Y positions of each fly’s centroid were automatically tracked and recorded with software custom written in LabView (National Instruments, USA) (Buchanan et al., 2015). We recorded the turning behavior of 3–6-day old flies, the standard age for measuring this behavior, for 2 h. Data from the small portion of individuals making fewer than 30 turns were discarded. Each fly was used only once.

To quantify turning predictability (the variability in turning bias across individuals), we computed the MAD, the median of the absolute deviation from each observation’s median (Buchanan et al., 2015), a metric of variability that is robust to outliers. We estimated MAD for each experimental group.

### Survival tests

We tested six experimental groups (2 spider conditions × 3 drug conditions): (1) fruit fly males grown without *P. apacheanus* spiders and without any drugs, (2) male flies grown without spiders on food supplemented with 5-HTP, (3) male flies grown without spiders on food supplemented with αMW, (4) male flies grown together with spiders on food without any drugs supplemented, (5) males raised with spiders on food supplemented with 5-HTP, (6) males grown together with spiders on food supplemented with αMW (Figure 1B). Upon eclosion, adult F1 flies were assayed on days 4–5.

To measure survival, we used ten Plexiglas jars (10 cm height × 12 cm diameter) and placed ten fruit flies of an experimental group into each jar (Figure 1B). Thus, we had ten jars for each survival group (48 jars containing 480 fruit flies) for 12 h during daylight time (Krams et al., 2016). We did not use carbon dioxide anesthesia to move fruit flies from their stock vials to survival jars. During survival tests, we placed one young (c. 6–7 months old) *P. apacheanus* spider and one vial containing fruit fly food into each plastic jar (Figure 1B). The spiders had access to water in a polycarbonate dish and a fitted luffa sponge. The spiders were deprived of food for c. 10 h before survival tests. Each spider was used only once.

### Statistics

To compare behavioral MADs across experimental groups, we used the permutation test. The data table of the proportion of right turns taken was shuffled, and the obtained MAD scores among randomized groups were compared to those of unshuffled data. The procedure was repeated 99,999 times, and *P*-values were calculated as the proportion of instances when the shuffled difference between group pairs was larger than the unshuffled difference. We performed a two-way ANOVA to assess the effect of development conditions and drugs added to the food on the subsequent survival of adult flies under predation risk. Tukey’s honest significance test followed the analysis. Turn bias (proportion of right turns) was compared between groups using Kruskal–Wallis Test by ranks. One Sample Wilcoxon Signed Rank Test was used to assess turn bias deviation from equal amount of right and left turns. We also compared the number of turns taken by fruit flies in the y-maze per minute using the Mann–Whitney *U* test.

Data analyses were performed in the R environment (version 4.1.0) (R Core Team, 2021). *P*-values of multiple comparisons were adjusted using Benjamini–Hochberg procedure (Benjamini and Hochberg, 1995). All differences were considered statistically significant when *P* < 0.05.

## Results

### Variability of turning behavior

**FIGURE 2:**
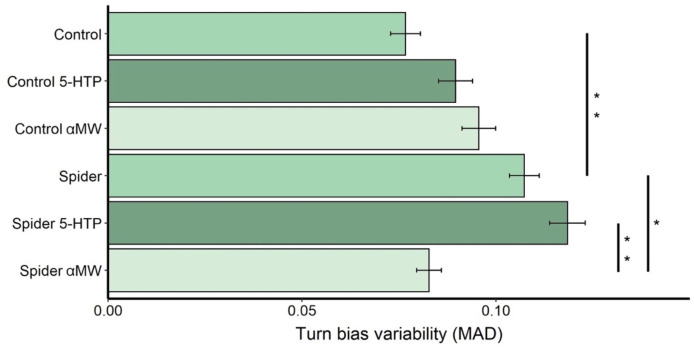
Turn bias variability (MAD) of fruit flies reared with and without spiders receiving different drug treatments. Error bars are ± SE estimated by bootstrap resampling. Asterisks indicate significant differences according to permutation tests: *0.05 > *P* > 0.01; **0.01 > *P* > 0.001.

Turn bias variability of male fruit flies grown with spiders (MAD = 0.11, *N* = 153 flies) was significantly higher than that of control flies (MAD = 0.08, *N* = 143) grown in a predator-free environment (Permutation test: *P* = 0.006; Figure 2). Feeding 5-HT to flies reared with spiders (MAD = 0.12, *N* = 116) did not increase the turning variability (*P* = 0.34) while feeding these flies αMW (MAD = 0.10, *N* = 140) significantly decreased turn bias variability (*P* = 0.021; Figure 1). Feeding 5-HTP (*P* = 0.33) and αMW (*P* = 0.12) did not affect the variability of turning behavior of control fruit flies (Figure 2).

### Handedness and the number of turns in the y-maze

**FIGURE 3:**
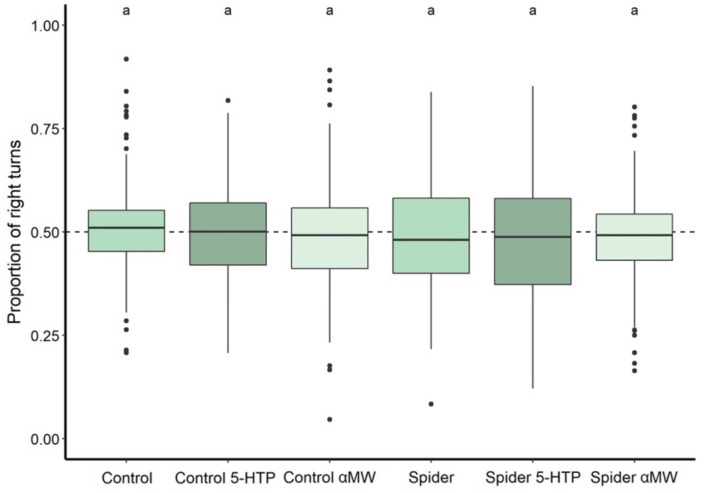
Turn bias of fruit flies reared with and without spiders and receiving different drug treatments during development. Thick lines indicate the median, and boxes indicate the 25th and 75th percentile. A dashed horizontal line indicates 0.5 proportion of right turns, a level at which flies take an equal amount of left and right turns. Thick lines indicate the median, boxes show the Q1 and Q3 quartiles, and whiskers represent the upper and lower quartile, excluding outliers. Black dots represent outliers: data points more than 1.5 times interquartile range away from Q1 and Q3. Experimental groups that are not statistically significantly different (Wilcoxon tests, *P* > 0.05) are indicated by the same letter at the top of the figure.

The proportion of the right turns (turn bias) did not differ among the groups of flies (Kruskal–Wallis: χ2 = 6.41, *P* = 0.268; Figure 3). Proportion of right turns by each group was also not significantly different from 0.5 (Wilcoxon tests: all *Ps* > 0.05; Figure 3), i.e., an equal number of right and left turns in each group.

**FIGURE 4:**
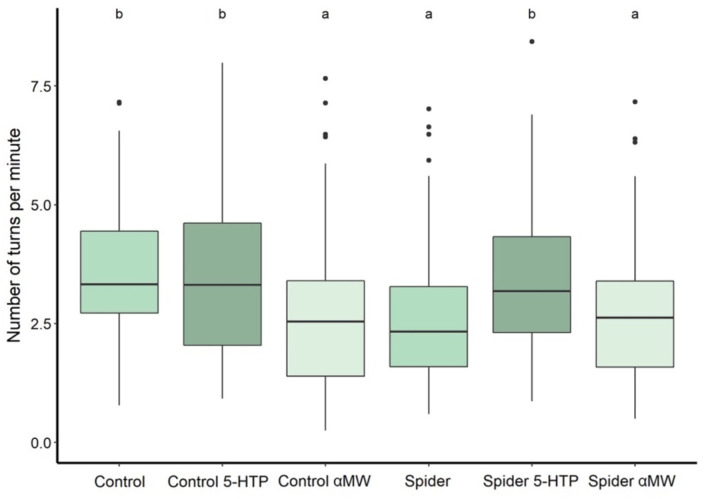
Turn rate (turns/minute) in the Y-maze of flies reared with and without spiders receiving different drug treatments. The flies reared with predators were previously exposed to predation during the larval stage, while in the control group, the flies were raised without jumping spiders. Thick lines indicate the median, boxes show the Q1 and Q3 quartiles, and whiskers represent the upper and lower quartile, excluding outliers. Black dots represent outliers (data points more than 1.5 times interquartile range away from Q1 and Q3). Experimental groups that are not statistically significantly different (Mann–Whitney tests, *P* > 0.05) are indicated by the same letter at the top of the figure.

Flies reared with spiders made significantly fewer turns per unit time (2.6 ± SD 1.3 turns/min) in the Y-maze compared to control flies (3.4 ± 1.5 turns/minute) (Mann–Whitney test: *P* = 0.0001; Figure 4). Feeding 5-HTP to flies reared with spiders significantly increased the turn rate (3.4 ± 1.4 turns/min) (*P* < 0.0001), whereas feeding them αMW had no significant effect (2.7 ± 1.3 turns/min) (*P* = 0.50; Figure 4). Feeding αMW to control flies significantly decreased the turn rate (2.6 ± 1.51 turns/min) (*P* = 0.0003), whereas feeding them 5-HTP had no significant effect (3.5 ± 1.71 turns/min) (*P* = 0.94; Figure 4).

### Survival

**FIGURE 5:**
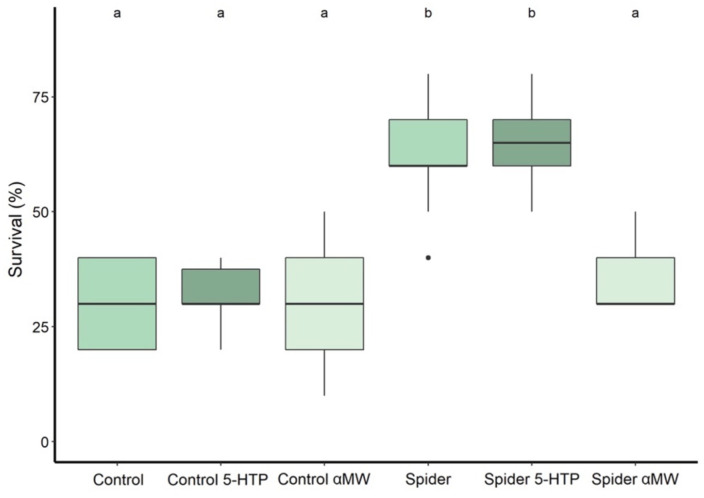
Survival percentage of adult fruit flies during a 12-h exposure to predation by jumping spiders. The flies reared with predators were exposed to predation during the larval stage; flies in the control group were raised without jumping spiders. Thick lines indicate the median, boxes show the Q1 and Q3 quartiles, and whiskers represent the upper and lower quartile, excluding outliers. Black dots represent outliers: data points more than 1.5 times interquartile range away from Q1 and Q3. Experimental groups that are not statistically significantly different (Tukey HSD, *P* > 0.05) are indicated by the same letter at the top of the figure.

When exposing adult flies to predation for 12 h, their survival was significantly affected by predator presence during the larval development (two-way ANOVA: *F*_1,54_ = 81.37, *P* < 0.0001), drug treatment (*F*_2,54_ = 14.76, *P* < 0.0001), and an interaction of both those factors (*F*_2,54_ = 12.57, *P* < 0.0001). Significantly more flies survived if they were reared under predator presence (mean survival: 62% ± SD 11.4%, *N* = 10) compared to the control group (30 ± 9.4%, *N* = 10) (Tukey HSD: *P* < 0.0001; Figure 5). Feeding flies reared with predators 5-HTP did not significantly affect their survival (65 ± 8.5%, *N* = 10) (*P* = 0.985; Figure 5), while feeding αMW significantly decreased their survival (35 ± 7.1%) (*P* < 0.0001; Figure 5). Feeding 5-HTP (32 ± 6.3%, *N* = 10) (*P* = 0.998; Figure 5) or αMW (30 ± 15%, *N* = 10) (*P* = 1.00; Figure 5) did not significantly affect the survival of flies of the control group.

## Discussion

The presence of predators is known to alter prey morphology (McCollum and Leimberger, 1997; Hossie et al., 2010) and exert selective pressure on prey escape ability (O’Steen et al., 2002; Krams et al., 2016; Janssens and Stoks, 2018). In this study, we found that the turning choices of fruit flies grown with predators are less predictable than that of flies grown in a predator-free environment. We also show that flies raised with predators survived under predation by spiders significantly better than flies grown without predators. Our results suggest that the higher variability/lower predictability of turning behavior of flies grown with predators may make them better at evading predation. We also show with pharmacological experiments that the effects of predator-rearing on turning variability and survival of *D. melanogaster* are regulated by the neurotransmitter serotonin, which also regulates the variability of turning behavior (de Bivort et al., 2022). However, these serotonin-associated effects applied only for fruit flies grown with spiders.

Unpredictable and erratic turning behavior in some animals makes them more challenging to attack (Yager et al., 1990; Bilecenoğlu, 2005; Eifler and Eifler, 2014), as is seen in both experimental (Jones et al., 2011) and modeling (Richardson et al., 2018) studies. Individual insects can exhibit substantial differences in escape behaviors, even in the absence of genetic variation (Schuett et al., 2011). Our results suggest a link between less predictable turning behavior and better survival under predation risk by jumping spiders that are sit-and-wait predators. One explanation is that growing up with predators provides prey with signals that are not generated by transient contact with predators post-development. Perhaps the effect of these signals is mediated by serotonergic neuromodulation during prey development. This idea is consistent with the observation that flies fed αMW during development, but without predators present, showed similar adult survival in the presence of spiders as control flies, suggesting that fruit fly individuality is not solely driven by 5-HT (Maloney, 2021).

Some previous work has shown that fruit flies reared in identical lab environments show broad diversity in their phototactic choices, variability which is under the control of 5-HT (Kain et al., 2012; Krams I. A. et al., 2021). Notably, inhibiting 5-HT synthesis was associated with higher phototactic variability — here we observed that inhibiting 5-HT reduced the excess turn bias variability seen in flies reared with spiders. Geographic variation of fruit fly phototaxis was consistent with a negative relationship between 5-HT and variability of phototactic choices. Flies from northern climates grow on food relatively deficient in the metabolic precursors of serotonin and had lower predictability of phototactic choices (Krams I. A. et al., 2021). Thus, the association between 5-HT and behavioral predictability went in opposite directions in the present study and previous work examining phototaxis. These contradictory results suggest that the control of 5-HT over different behaviors may lead to different results, probably because different serotonin-responsive neuronal circuits are involved in different behaviors. To better understand the developmental, epigenetic and neurophysiological changes caused by direct predation and non-lethal predator presence, more study of behavior-specific neurobiological effects is required.

Our results support the results by Pantoja et al. (2016) examining variability in zebrafish (*Danio rerio*) antipredator locomotor behaviors. They found that zebrafish individuals show significant variation in acoustic startle responses. These responses are linked with the neurosecretion of dorsal raphe neurons (Pantoja et al., 2016). It was shown that zebrafish individuals show a higher fraction of serotonergic dorsal raphe nucleus neurons active during predator attacks. Pantoja et al. (2016) also showed that heightened 5-HT prevented habituation to predator stimuli, which improves the efficiency of antipredator behavior and survival of the prey. Together, these results suggest the importance of serotonergic signaling in the CNS and its ontogenetic development in establishing a distribution of antipredator behaviors across individuals.

The results of this study may have evolutionary implications. It is known that without phenotypic variation, there would be no evolution by natural selection. However, we show that individuals with similar genotypes raised in similar environments, except for the presence/absence of spiders, may significantly differ in their simple behavioral reactions, (such as left vs. right decision in the absence of an asymmetric stimulus in the Y-maze). This suggests that asymmetries within the brain predispose the animal to go one way rather than the other and that neural activity influences the variation between animals (Buchanan et al., 2015). As these predispositions are relatively stable within individuals with considerable among-individual differences in behaviors (Réale et al., 2010; Buchanan et al., 2015; Roche et al., 2016; Trakimas et al., 2019), behavioral reactions of this kind are coined animal personality. Our results show that fruit flies may use a simple mechanism to dynamically regulate their behavioral individuality with individual variation in wiring and behavior as a general feature of neural circuits to facilitate individual adaptations and survive in changing environments (Mollá-Albaladejo and Sánchez-Alcañiz, 2021). However, explaining the proximate origins of changes in behavioral variability as a response to environmental challenges is not easy. Behavioral phenotypes emerge from many different levels of biological organization, including sensing of predators in the environment, adaptive gene expression, and even stochasticity in gene expression (Raj et al., 2010; Li et al., 2017; Honegger and de Bivort, 2018) to develop biases in idiosyncratic behavioral responses (Werkhoven et al., 2021) without changes in average left-right turning preferences.

This study found that flies reared with spiders were less mobile than control flies. Our recent study shows that predator stress during larval development of Drosophila impairs carbohydrate metabolism by systemic inhibition of Akt protein kinase, which is a central regulator of glucose uptake (Krama et al., revision 2, personal observation). This metabolic disorder is a likely cause of developing a diabetes-like biochemical and behavioral phenotype. An inability to metabolize glucose shifts the metabolism of fruit flies to triglyceride consumption, which decreases walking activity and might be a direct reason for the enhanced survival of fruit flies grown with spiders. Consistent with this idea, carbohydrate metabolism was found as one of the molecular functions most enriched in genes whose expression variation predicts variation in locomotor activity among individual isogenic flies (Werkhoven et al., 2021). However, the mechanism causing the higher variability of the turning behavior in flies with a diabetes-like phenotype remains unknown.

Antipredator behavior consists of a complex set of behavioral and physiological reactions and therefore likely involves neural pathways other than 5-HT. Honegger et al. (2020) found that both 5-HT and dopamine affect olfactory preference variability in fruit flies, and it is known that fruit flies can detect predators by their odors (Krams R. et al., 2021). Omura et al. (2012) and Stern et al. (2017) showed that the roaming speed of animals might depend on such neurotransmitters as tyramine, octopamine, npr-1, and daf-7, in addition to 5-HT. This suggests that future research on the neural regulation of antipredator responses in fruit flies should examine the effects of several neurotransmitters and their possible interactions. Experimental manipulations targeting more than one neuromodulator may be essential, as one neuromodulator can alter the efficacy of other neuromodulators (Niederkofler et al., 2015; Niens et al., 2017). Finally, animals may respond to neuromodulators differentially based on their personalities (Krams et al., 2018). The complex interactions of neuromodulators and their behavior-specific effects on predictability will make this a rich and challenging area of research.

## Data availability statement

The data that supports the findings of this study are available from the following Zenodo repository: https://zenodo.org/search?page=1&size=20&q=7936563.

## Author contributions

TK, RK, and BB conceived and designed the study. TK, MM, RK, GT, SP, BB, and IK performed the study and collected and extracted data. MM, DE, GT, SP, and IK analyzed the data. TK, RK, TG, PJ, and KZ maintained stocks of experimental flies and spiders. ES, BB, and IK built the equipment. TK, MM, BB, and IK wrote the manuscript. TK, MM, RK, TG, GT, PJ, SP, KZ, DE, MR, ES, JC-G, BB, and IK participated in data analyses, results interpretation, and drafting the manuscript. All authors contributed to the article and approved the submitted version.
