# Corrigendum: Development under predation risk increases serotonin-signaling, variability of turning behavior and survival in adult fruit flies *Drosophila melanogaster*

**DOI:** 10.3389/fnbeh.2024.1391782

**Published:** 2024-03-22

**Authors:** Tatjana Krama, Māris Munkevics, Ronalds Krams, Tatjana Grigorjeva, Giedrius Trakimas, Priit Jõers, Sergejs Popovs, Krists Zants, Didzis Elferts, Markus J. Rantala, Eriks Sledevskis, Jorge Contreras-Garduño, Benjamin L. de Bivort, Indrikis A. Krams

**Affiliations:** ^1^Department of Biotechnology, Institute of Life Sciences and Technologies, Daugavpils University, Daugavpils, Latvia; ^2^Chair of Plant Health, Estonian University of Life Sciences, Tartu, Estonia; ^3^Department of Zoology and Animal Ecology, Faculty of Biology, University of Latvia, Riga, Latvia; ^4^Institute of Biosciences, Vilnius University, Vilnius, Lithuania; ^5^Institute of Molecular and Cell Biology, University of Tartu, Tartu, Estonia; ^6^Department of Botany and Ecology, Faculty of Biology, University of Latvia, Riga, Latvia; ^7^Department of Biology, Turku Brain and Mind Center, University of Turku, Turku, Finland; ^8^Department of Technology, Institute of Life Sciences and Technologies, Daugavpils University, Daugavpils, Latvia; ^9^Escuela Nacional de Estudios Superiores, Universidad Nacional Autónoma de México, Morelia, Mexico; ^10^Institute for Evolution and Biodiversity, University of Münster, Münster, Germany; ^11^Department of Organismic and Evolutionary Biology, Harvard University, Cambridge, MA, United States; ^12^Latvian Biomedical Research and Study Centre, Riga, Latvia; ^13^Institute of Ecology and Earth Sciences, University of Tartu, Tartu, Estonia; ^14^Department of Psychology, University of Tennessee, Knoxville, Knoxville, TN, United States

**Keywords:** *Drosophila melanogaster*, behavioral predictability, serotonin, survival under predation, turning behavior

In the published article, there was an error: five sentences are missing.

A correction has been made to Materials and methods, “*Prey, predators, developmental speed, and the main treatment groups*,” the very end of the 4th paragraph. After this sentence: “The density of F1 first-instar larvae across the vials was similar, and we averaged the density to 120 larvae/vial by removing extra larvae with a squirrel brush (Krams et al., 2016),” there should be five more sentences.

The corrected paragraph appears below:

“We isolated fruit flies using carbon dioxide anesthesia within 6–7 h after eclosion. Ten F0 females and ten males were placed for 24 h in one vial (Flystuff polystyrene vials; Genesee Scientific, El Cajon, CA, USA, 24 mm inner diameter × 95 mm height) containing 6 ml of Cal Tech medium. After 24 h, the adults were removed, and the vials were placed horizontally on the floor of Plexiglas jars (10 cm height × 12 cm diameter). The density of F1 first-instar larvae across the vials was similar, and we averaged the density to 120 larvae/vial by removing extra larvae with a squirrel brush (Krams et al., 2016). Vials with *Drosophila* larvae were randomly divided into two groups: one that was exposed to spiders and one that was not. In the spider-treated group, a single *P. apacheanus* individual was also included in each Plexiglas jar. The vials did not have stoppers, giving the spider free access to the developing flies (as well as the fly media). Developing flies were also exposed to the odor of the spider throughout the container. Flies for behavioral and survival assays were removed the day after they eclosed, without anesthesia, and transferred to drug-treated vials as described below.”

The authors apologize for this error and state that this does not change the scientific conclusions of the article in any way. The original article has been updated.
